# Fused Double Supernumerary Premolars of the Mandible: A Rare Case

**DOI:** 10.1155/2012/734670

**Published:** 2012-12-17

**Authors:** Ali Azhar Dawasaz, Syed Sadatullah, Syed Kamran Bokhari

**Affiliations:** ^1^Division of Oral Medicine and Radiology, College of Dentistry, King Khalid University, Abha, Saudi Arabia; ^2^Division of Oral Biology, College of Dentistry, King Khalid University, Abha, Saudi Arabia; ^3^Division of Oral Surgery, College of Dentistry, King Khalid University, Abha, Saudi Arabia

## Abstract

The incidence of nonsyndromic supernumerary premolars is rare. Supernumerary premolars are likely to undergo pathological changes. The most commonly encountered complications with these teeth are dentigerous cyst and root resorption of the adjacent tooth. This paper is about impacted double fused supernumerary premolars in the right mandiblular body associated with an impacted first premolar in a 17-year-old male. Under local anesthesia, the supernumerary premolars and the impacted permanent first premolar were surgically removed. Early diagnosis followed by an appropriate treatment at the right time will result in favorable prognosis in such cases.

## 1. Introduction

Supernumerary teeth (ST) occur in addition to the normal complement of teeth in permanent or deciduous dentitions [1]. These teeth may remain embedded in the alveolar bone or can erupt into the oral cavity. When they remain embedded, they may cause disturbance to the developing teeth [2]. It has been reported that the prevalence of supernumerary premolars (SP) is between 0.075 and 0.26% and that supernumerary premolars account for only 8–10% of all the supernumerary cases [3]. The difference between these teeth and the other supernumeraries is that they occur more commonly in the mandible [4]. SP usually occur as a solitary tooth. Single supernumeraries occur in 76–86% of cases and double supernumeraries occur in 12–23% of the cases [5].

ST is considered a developmental anomaly and has been argued to arise from multiple etiologies. Different factors give rise to different types of supernumeraries and combined etiological factors responsible for same. Some of the theories for the formation of ST include atavism, splitting of the tooth bud, local, independent conditioned hyperactivity of the dental lamina, and a combination of genetic and environmental factors [1, 6, 7]. However, the most accepted theory is regarding the hyperactivity of dental lamina [7]. According to this theory, the lingual extension of an additional tooth bud leads to a eumorphic tooth, while the rudimentary form arises from proliferation of epithelial remnants of the dental lamina induced by pressure of the complete dentition [8]. Late developing (postpermanent) supernumerary teeth develop from the proliferation of the dental lamina after the permanent dentition is completed [9].

ST are classified according to their morphology as rudimentary or supplemental. Rudimentary teeth are smaller and tuberculate in shape [10], whereas the term supplemental is used when the ST usually resemble the teeth of a group with which they are associated.

The presence of ST may be part of developmental disorders such as cleft lip and palate, cleidocranial dysostosis, Gardner's syndrome, Fabry Anderson's syndrome, Ellis-Van Creveld syndrome (chondroectodermal dysplasia), Ehlers Danlos syndrome, incontinentia pigmenti, and Tricho-rhino-phalangeal syndrome [11].

This paper aims to document a rare case of nonsyndromic fusion of two supernumerary supplemental premolars associated with an impacted permanent first premolar.

## 2. Case Description

**Figure 1:**
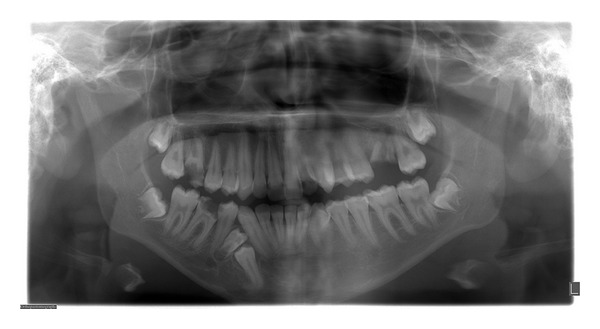
Orthopantomograph showing unilateral impacted partially formed and fused double premolars associated with, but separate from an impacted premolar.

**Figure 2:**
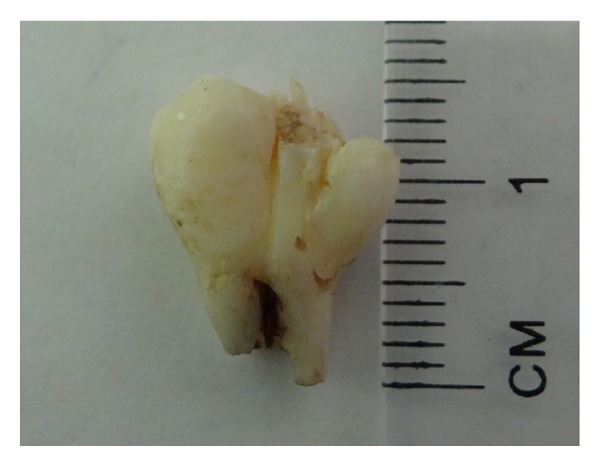
Extracted fused supernumerary premolars.

**Figure 3:**
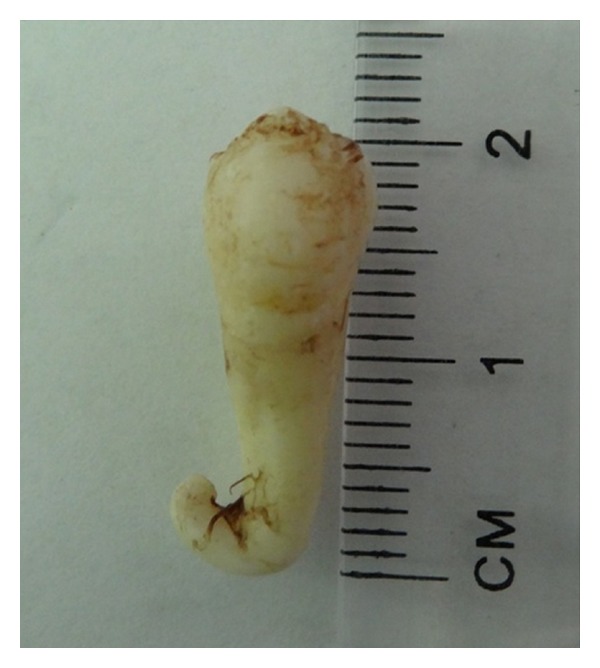
Extracted premolar with severe dilacerated root.

**Figure 4:**
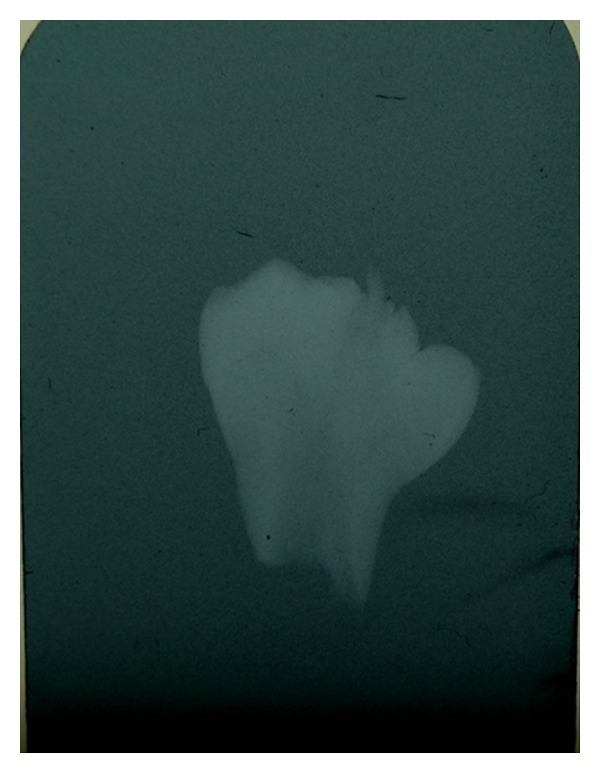
Radiograph showing two separate pulp chambers and root canals of extracted fused supernumerary teeth.

**Figure 5:**
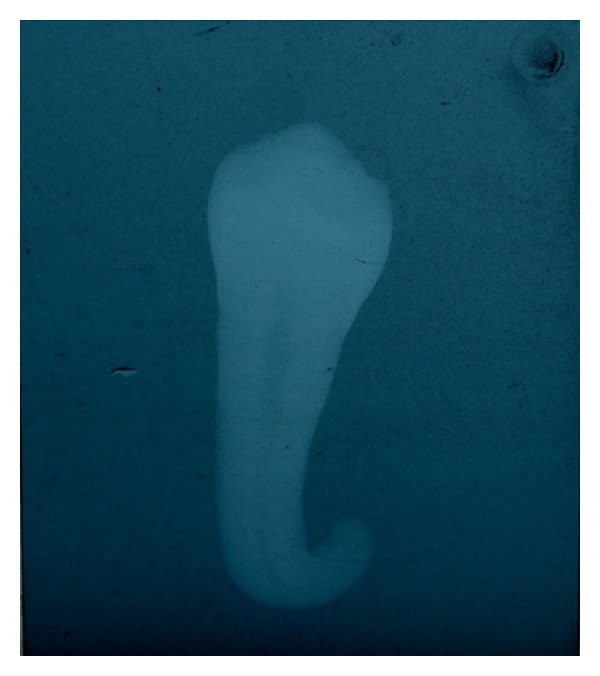
Radiograph of impacted premolar.

A 17-year-old Saudi male visited the Dental Diagnosis Clinic of King Khalid University, Abha, Saudi Arabia, with a chief complaint of pain in lower right jaw region. No extra oral abnormality was observed. Intraoral examination revealed a missing premolar in the lower right quadrant and tilted permanent right mandibular canine. Orthopantomograph revealed unilateral impacted partially formed and fused double premolars associated with, but separate from an impacted premolar (Figure 1). After taking patient consent and performing presurgical physical health status evaluation, it was decided to extract both the supernumerary and impacted premolars under local anesthesia. Bone was removed using slow-speed bur with copious saline irrigation. The structures were successfully removed in total (Figures 2 and 3). The margins of the bone were smoothened and absorbable gelatin sponge (Gelfoam, Pharmacia, Zuellig) placed in the socket. The flap was sutured with Coated Vicryl 4/0 (Ethicon, Inc., Johnson and Johnson Company, USA) and haemostasis was achieved. The postoperative course was uneventful. Radiographic evaluation of the extracted fused supernumerary teeth revealed two separate pulp chambers and root canals (Figure 4). A severely dilacerated root of the impacted premolar was also observed (Figure 5). The patient was referred for orthodontic assessment to correct the malposed permanent right mandibular canine.

## 3. Discussion

The prevalence of SP has been reported differently in various studies due to the differences in patient population samples, age groups, ethnicity, and applied radiographic techniques [1]. SP are said to represent between 8% [12] and 9.1% [13] of all supernumerary teeth. Although, literature reports increased occurrence of the supernumeraries in the maxilla [14], paramolar, distomolar, and supernumerary premolars are more likely to develop in the mandible [15]. SP usually resemble premolars that are normal in shape and size. In a study by Salcido-García et al. [16], the prevalence of SP was found to be 1.7% whereas another study from USA, in which 1100 orthodontic patients were included, SP was found to be 0.64% [17]. In yet another study, Esenlik et al. [18] reported the prevalence of the maxillary SP to be 0.2% and mandibular SP to be 0.5%. Approximately 75% of SP becomes impacted [1, 6]. SP occur more frequently in males than females [6].

ST may occur with or without more than 20 syndromes and developmental conditions; however, nonsyndromic multiple supernumerary teeth are rarely encountered prevalence of this being 0.08% [7, 16, 19].

SP are usually asymptomatic and most cases are diagnosed by chance during inspection of radiographs prior to the commencement of orthodontic treatment [1, 7]. Supernumeraries generally cause problems of malocclusion of local nature like tipping of adjacent teeth, rotation, bodily displacement, delayed eruption, or prevent eruption of tooth of normal series. Also, they may lead to esthetic disharmony and functional distortion [20]. Bodin et al. [19] have reported that only 2% of the supernumerary premolars are likely to undergo pathological changes. Nevertheless, the most commonly encountered complications with these teeth are dentigerous cyst and root resorption of the adjacent tooth [1].

Compression of SP on the adjacent teeth and their closeness to the mental and inferior dental nerves may lead to pain [21]. Our patient complained of pain in the impacted premolar region, which could have been due to pressure and proximity of SP to the inferior dental nerve as evident in the orthopantomograph (Figure 1).

Tooth fusion is defined as union between the dentin and/or enamel of two or more separate developing teeth [22]. Shafer et al. [23] proposed that pressure produced by physical force prolongs the contact of the developing teeth causing fusion. Lowell and Soloman [24] believe that fused teeth result from physical action that causes the young tooth germs to come into contact, thus producing necrosis of the intervening tissue and allowing the enamel organ and dental papilla to fuse together. Many authors have also suggested an autosomal dominant trait with reduced penetrance to be the cause [25]. Fusion may occur between two normal teeth or between a normal tooth and a supernumerary tooth. Radiographically, the dentin of fused teeth always appears to be joined in some region with separate pulp chambers and canals. Most authors agree that there is no sex difference and location of the malformation. It is usually restricted to the canine-incisor region. The frequency of fused teeth is estimated to be 0.5% in the primary dentition and 0.1% percent in the permanent dentition [26, 27]. Structurally there is always a union between the dentin of the fused tooth which can vary from partial to complete fusion of both roots and crowns. Consequently, pulp chambers may be separated or common to both teeth [27]. Radiographic examination is necessary to reach correct diagnosis.

In our paper a short root and one root canal were clearly visible on the radiograph of the SPs. Due to the crown form and short root, the authors of this paper diagnosed it as fusion of the two supernumerary teeth. This represents a rare combination of fusion of two supernumerary supplemental premolars. These fused SPs have also caused impaction of the neighbouring second premolar. Treatment options available in such cases include (1) Maintenance in situ with regular followup; (2) extraction if accompanied by pathologic changes; (3) orthodontic treatment.

Treatment decision should involve judicious assessment of the case considering the potential risks of leaving ST in situ or the hazards of surgical removal. The consequences of surgical removal of impacted ST, especially in the mandibular premolar region should be evaluated, where the teeth are in close proximity to the inferior dental and mental nerves [28]. The timing of surgical removal is as much debated among clinicians as are the treatment methods [1]. SPs begin their calcification late; hence, their complete development takes more time as compared to normal teeth [1, 29]. Therefore, it is advisable to postpone surgical intervention until the end of the development of permanent dentition.

In cases when these teeth are associated with pathological formation or when they hinder the eruption of, or give rise to malpositioning of permanent teeth, they should be removed at the earliest [16]. In our case, the ST were extracted immediately to correct this functional distortion and relieve the patient from pain. There was relief from pain reported after extraction, vindicating the perception that mandibular impacted SPs can cause compression of inferior dental nerve leading to pain.

## 4. Conclusion

Early diagnosis and proper selection of treatment method is likely to result in favorable prognosis. In order to provide symptomatic relief and avoid pathological complications, we deduce that surgical intervention of impacted fused SPs is the most appropriate treatment method.
